# Molecular Docking and 3D-Pharmacophore Modeling to Study the Interactions of Chalcone Derivatives with Estrogen Receptor Alpha

**DOI:** 10.3390/ph10040081

**Published:** 2017-10-16

**Authors:** Muchtaridi Muchtaridi, Hasna Nur Syahidah, Anas Subarnas, Muhammad Yusuf, Sharon D. Bryant, Thierry Langer

**Affiliations:** 1 Department of Pharmaceutical Analysis and Medicinal Chemistry, Faculty of Pharmacy, Universitas Padjadjaran, Jln. Raya Bandung Sumedang KM. 21, Jatinangor 45363, West Java, Indonesia; hasnans20@gmail.com; 2 Department of Pharmacology, Faculty of Pharmacy, Universitas Padjadjaran, Jln. Raya Bandung-Sumedang KM. 21, Jatinangor 45363, West Java, Indonesia; aasubarnas@yahoo.co.id; 3 Department of Chemistry, Faculty of Mathematics and Natural Sciences, Universitas Padjadjaran, Jln. Raya Bandung-Sumedang KM. 21, Jatinangor 45363, West Java, Indonesia; m.yusuf@unpad.ac.id; 4 Inte: Ligand GmbH, Mariahilferstrasse 74B/11, A-1070 Vienna, Austria; bryant@inteligand.com; 5 Department of Pharmaceutical Chemistry, Faculty of Life Sciences, University of Vienna, Althanstraße 14, A-1090 Vienna, Austria; thierry.langer@univie.ac.at

**Keywords:** chalcone, molecular docking, structure-based 3D pharmacophore modeling, anti-breast cancer, estrogen receptor α, MCF-7

## Abstract

Tamoxifen is the most frequently used anti-estrogen adjuvant treatment for estrogen receptor-positive breast cancer. However, it is associated with an increased risk of several serious side–effects, such as uterine cancer, stroke, and pulmonary embolism. The 2′,4′-dihydroxy-6-methoxy-3,5-dimethylchalcone (ChalcEA) from plant leaves of *Eugenia aquea*, has been found to inhibit the proliferation of MCF-7 human breast cancer cells in a dose-dependent manner, with an IC_50_ of 74.5 μg/mL (250 μM). The aim of this work was to study the molecular interactions of new ChalcEA derivatives formed with the Estrogen Receptor α (ERα) using computer aided drug design approaches. Molecular docking using Autodock 4.2 was employed to explore the modes of binding of ChalcEA derivatives with ERα. The 3D structure-based pharmacophore model was derived using LigandScout 4.1 Advanced to investigate the important chemical interactions of the ERα-tamoxifen complex structure. The binding energy and the tamoxifen-pharmacophore fit score of the best ChalcEA derivative (HNS10) were −12.33 kcal/mol and 67.07 kcal/mol, respectively. The HNS10 interacted with Leu346, Thr347, Leu349, Ala350, Glu353, Leu387, Met388, Leu391, Arg394, Met421, and Leu525. These results suggest that the new ChalcEA derivatives could serve as the lead compound for potent ERα inhibitor in the fight against breast cancer.

## 1. Introduction

Breast cancer accounts for more than 1.2 million new cases and 500,000 deaths annually. It is the leading cause of cancer death among females (23% of total cancer and 14% of cancer mortality cases) [1]. Mortality in women due to this cancer in both developed (189,000 deaths) and emerging (269,000 deaths) economies was reported to reach 15.5% and 12.7% of total breast cancer cases, respectively in 2012 [2]. In 2013, the prevalence of breast cancer in Indonesia was reported to afflict 1.4% of the total population (approximately 347,792 persons) [3].

Estrogen plays a critical role in the growth and development of bone, breast, and uterine pathology. There are two subtypes of estrogen receptor, ERα (Estrogen Receptor-α) and ERβ (Estrogen Receptor-β). ERα plays a role in cell proliferation and has been found in the endometrial, breast cancer and ovarian stromal cells, as well as in the hypothalamus [4]. The most commonly used anti-estrogen adjuvant treatment for ERα-positive (ERα+) premenopausal women is tamoxifen, a selective estrogen receptor modulator (SERM). The active metabolite of tamoxifen is 4-hydroxytamoxifen (4-OHT) [5]. Tamoxifen is also often prescribed for postmenopausal patients with ERα+ tumors. It acts as an antagonist to ERα and inhibits its signaling pathway in ERα+ breast cancer cells. Tamoxifen therapy significantly reduces the risk of breast cancer recurrence. The tamoxifen-bound ER complex inhibits the genes from being switched on by estrogen, leading to the prevention of the estrogenic effects responsible for cancer cell proliferation [6].

Despite the obvious benefit of tamoxifen in breast cancer treatment, this drug also has serious risks. For example, the risk of endometrial malignancy and hyperplasia varies from 1.5- to 6.9-fold [7] after cumulative and long duration usage [8] due to its agonistic effect in the uterus. In addition, the potential for endometrial cancer increased significantly with overweight postmenopausal females. To further complicate matters, many ER+ patients, regardless of high levels of ER, demonstrated intrinsic resistance to hormonal therapies. Thus, alternative treatments are needed.

One promising natural anti-breast cancer adjuvant compound is chalcone, a flavonoid group secondary metabolite found in many plants. The 2′,4′-dihydroxy-6-methoxy-3,5-dimethylchalcone (ChalcEA) from the leaves of *Eugenia aquea* inhibited the cell proliferation of MCF-7 human breast cells, in a dose-dependent manner, with an IC_50_ of 74.5 μg/mL (250 μM). It has been hypothesized to promote apoptosis via the activation of poly(adenosine diphosphate-ribose)polymerase (PARP). Further investigations have provided a basis for its use in breast cancer disease management [7]. However, its IC_50_ value was considered as a moderate level of inhibition compared to tamoxifen [8]. Therefore, further efforts to improve the inhibitory activity of ChalcEA through structural modifications guided by computer-aided drug design (CADD) methodologies, such as molecular docking method and 3D-pharmacophore modeling, were explored in this study.

The biological activity of a compound defined by the affinity of a small-molecule ligand towards the macromolecular receptor can be explored using in silico methods and compared to the experimental methods. Molecular docking scoring functions can compute binding affinities that are useful for predicting bioactive poses of compounds and prioritizing compounds for experimental assessment. These structure-based drug design methods are useful for drug discovery research. The molecular docking approach can be used to model an interaction between a small molecule (ligand) and protein at the atomic level. The docking process involves two basic steps: prediction of multiple structural conformations in a binding pocket (pose), and scoring the pose in order to rank the multiple solutions [9].

A pharmacophore represents the key interaction features of a molecule responsible for eliciting or blocking biological activity [10]. 3D-pharmacophore models not only describe the chemical feature types but also define the 3D geometry of the features of a bioactive compound. Ligand-based (LB) pharmacophore modeling involves a set of known active molecules without including information from the macromolecular target. Structure-based (SB) pharmacophore models are composed of features derived from interactions between the binding pocket and the ligand and require a target ligand complex binding pocket. Pharmacophore models resulting from SB and LB approaches are used to understand the key interaction features of a set of active molecules and how a bioactive ligand interacts with the target-binding site. The 3D-models can be used to search for bioactive molecules using virtual screening methods or to provide medicinal chemistry decision support during hit expansion and lead optimization [11]. The pharmacophore modeling study involved the evaluation of the key pharmacophore interaction features of 4-OHT, ChalcEA and the derivatives reported herein.

## 2. Results

**Figure 1:**
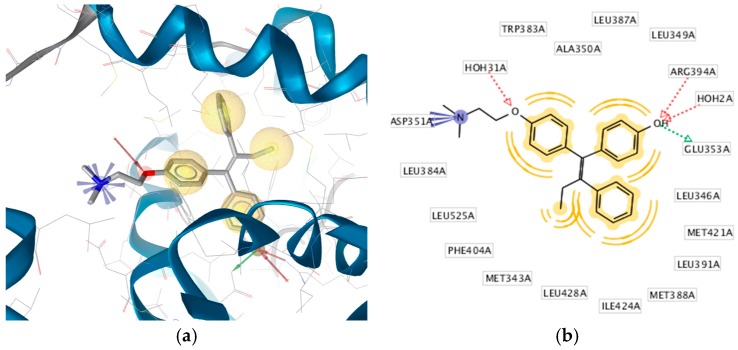
(**a**) Pharmacophore-Molecular Docking Based of 4-OHT with ERα derived from the X-ray derived structure (PDB code: 3ERT). Hydrophobic, positive ionizable, hydrogen bond donor and acceptor interactions are depicted as yellow spheres, blue star, green and red arrows, respectively. (**b**) The 2D-depiction illustrates a hydrophobic pocket with hydrophobic interactions with the binding site residues. Interactions derived and depicted using LigandScout 4.1 Advanced. Hydrogen atoms on the ligand and excluded volumes (restricted areas that define the shape of the binding pocket) are not displayed.

The X-ray derived structure of ERα in complex with 4-OHT (PDB code: 3ERT) was selected for molecular docking studies of ChalcEA due to good parameters for experimental resolution (1.9 Å), R-value free and R-value work of 0.262 and 0.229, respectively [12]. The interactions derived from the X-ray derived structure revealed that 4-OHT formed hydrophobic interactions predominantly with the butenyl group and aromatic rings, a positive ionizable interaction with primary the tertiary amine nitrogen and hydrogen bond interactions with the phenoxy and hydroxyl oxygens (Figure 1a,b).

The ligand binding domain (LBD) of ERα is mainly a hydrophobic cavity composed of residues from helices 3, 6, 7, 8, 11 and 12 [13]. Helix-12 of the ER (residues 536–544) plays a major role in determining the agonist or antagonist activity of a ligand. When 4-OHT, an antagonist, binds to the ERα LBD, helix-12 occludes the co-activator recognition groove resulting in antagonist activity. This phenomenon occurs due to the absence of hydrogen bonding with His524 when 4-OHT is bound. In contrast, hydrogen bonding occurs with His524 and the ER agonist estradiol [13]. 

**Figure 2:**
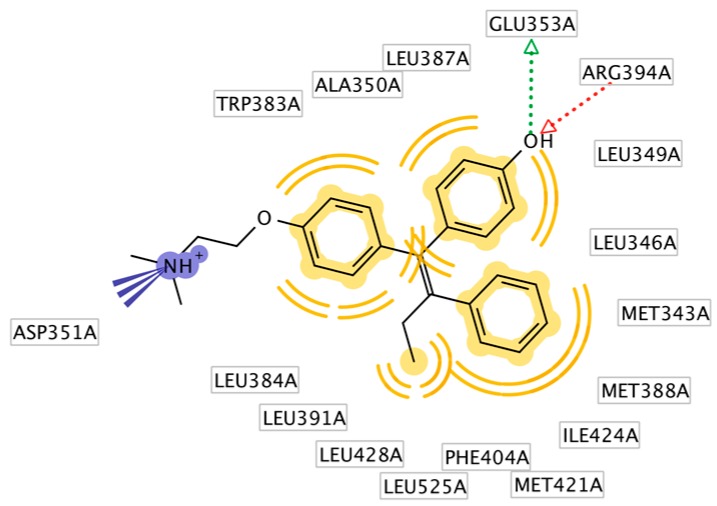
Best docked pose of 4-hydroxytamoxifen (4-OHT) with estrogen receptor-alpha (ERα) using AutoDock 4.2.

Before performing the molecular docking simulation, we first validated our docking method by extracting 4-OHT from the crystallographic ERα structure and docking it into the binding site to verify that the docking program could reproduce the antagonist bioactive conformation of 4-OHT. The best-docked ligand conformation shown in Figure 2 had a root mean square deviation (RMSD) of 0.893 Å compared to the original X-ray derived conformation.

### 2.1. Modification of ChalcEA Derivatives

**Figure 3:**
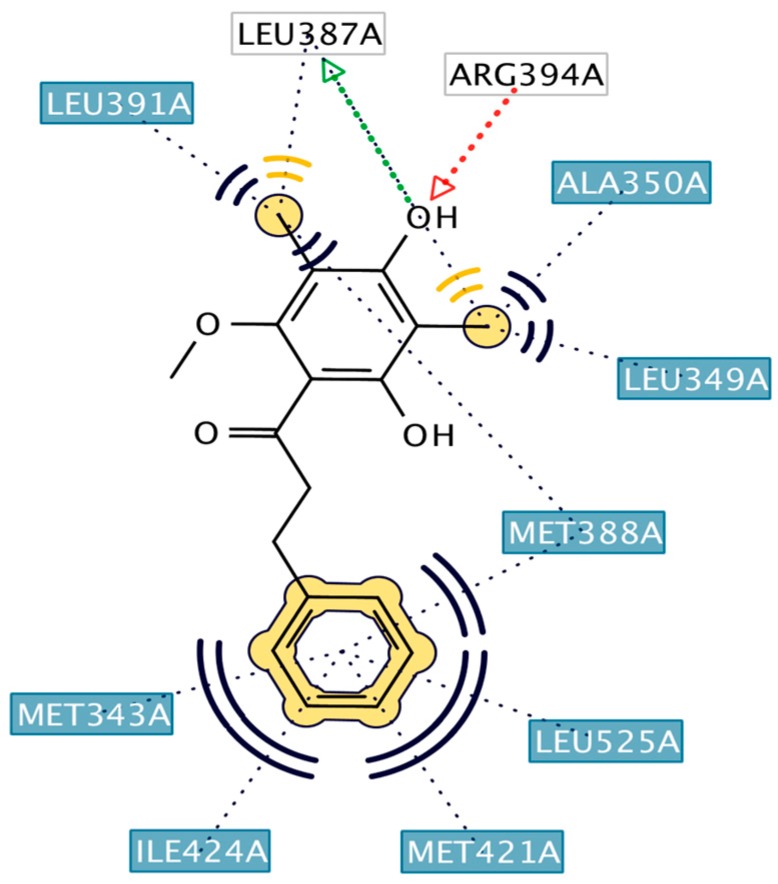
Best docked pose of ChalcEA with ERα. One hydrogen bond donor, one hydrogen acceptor and three hydrophobic (pi-alkyl) interactions are represented with green, red, and black, colored dashed lines, respectively. This interaction was visualized by LigandScout 4.1.

**Figure 4:**
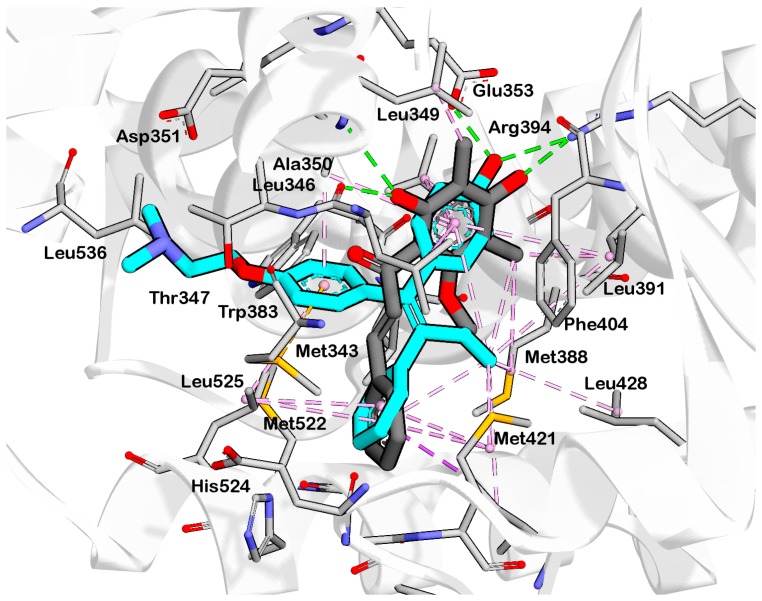
Overlay of the docked pose of ChalcEA (gray) and 4-hydroxy-tamoxifen (4-OHT) (blue) in the binding site of estrogen receptor alpha (ERα). Hydrogen bond and pi-alkyl interactions are represented in green and pink colored dashed lines, respectively.

The best-docked conformation of ChalcEA (Figure 3) within the LBD of ERα revealed hydrogen bonds with the 2′ and 4′ hydroxyl groups, whereas the carbonyl group did not form any interaction. Comparison of the predicted binding poses of ChalcEA and 4-OHT showed that two aromatic rings from each molecule were positioned similarly in the LBD (Figure 4). 

However, the dimethylaminoethoxy group of 4-OHT extended further than the carbonyl group of ChalcEA. This difference might account for the higher computed free energy of binding (∆G) of ChalcEA (−8.23 kcal/mol) compared to a ∆G of −11.04 kcal/mol for 4-OHT.

Therefore, the design of ten new ChalcEA derivatives shown in Table 1 focused on modifications at the position of the carbonyl group and dihydroxy substituted aromatic ring. All modifications were guided by the key ERα interactions with 4-OHT (Figure 1), and Lipinski’s Rule of Five (molecular weight less than 500, log P or coefficient partition between −5 and 5, less than five hydrogen bond donors, and less than ten hydrogen bond acceptors [14] (Table 2). Therefore, we aimed to increase the hydrophobicity around the dihydroxyl substituted aromatic ring, by replacing hydroxyl groups with methoxy groups and introducing extended substitutions at the R5 position of ChalcEA (Table 1).

The docking results of the ten derivatives, HNS1-HNS10 is summarized in Table 3.

The ∆G calculated the free energy of binding ranged from −12.33 to −9.30 kcal/mol. The best pose from each derivative exhibited better ∆G than the best-docked pose of ChalcEA (−8.23 kcal/mol). Seven out of ten ChalcEA derivatives formed a hydrogen bond with Arg394, similar to the 4-OHT.

### 2.2. Structure-Based 3D Pharmacophore Modeling

**Figure 5:**
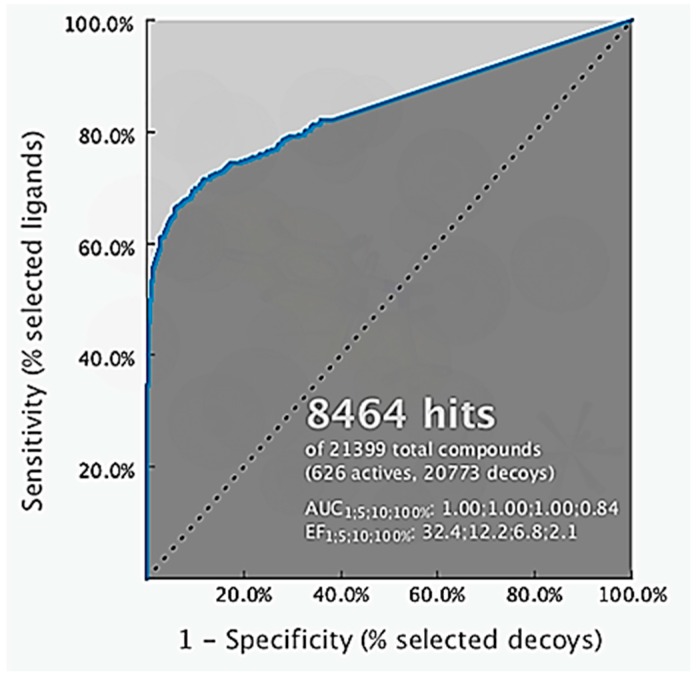
Receiver operating characteristic (ROC) curve validation of the 3D structure-based pharmacophore model using a set of 626 estrogen receptor alpha active and 20,773 decoy molecules.

The 3D structure-based pharmacophore model was created using LigandScout 4.1 Advanced. LigandScout automatically derives key chemical features, such as hydrogen bond donors and acceptors, hydrophobic, positive and negative ionizable, aromatic interactions along with their 3D-geometries of a bioactive molecule. During virtual screening, it aligns ligands based on their pharmacophore features in 3D space using an advanced algorithm [15]. The validation of the structure-based 3D pharmacophore model involved virtual screening of 626 molecules active at ERα and 20,773 ERα decoys taken from the enhanced Database of Useful Decoys (DUDe) using the derived 3D-model. The early enrichment factor (EF_1%_) was 32.4 with an excellent AUC (area under the ROC curve) value of 1.00 (Figure 5) indicating that the pharmacophore model was able to distinguish successfully true active from decoy ERα molecules in accordance with methods described by Kirchmair et al. [16]. The key pharmacophore interaction features of 4-OHT and ERα included hydrophobic, a positive ionizable and hydrogen bond donor and acceptors (Figure 1). Virtual screening results of ChalcEA the ten derivatives are reported in Table 4 in the form of a pharmacophore fit score which measures the geometric fit of the features of a molecule to the 3D-structure-based pharmacophore model. A higher fit score indicates a better fit to the model, and therefore molecules that fit to the pharmacophore model should also show activity at ERα because not all of the features of the model could be matched any two features could be omitted during the virtual screening process. In this case, features that could not be matched would result in lower pharmacophore fit scores. Interestingly, the pharmacophore fit scores of all of the derivatives (46.61 to 67.07) were all higher than the parent compound, ChalcEA (45.90). HNS9 and HNS10 had the best pharmacophore fit scores meaning that their chemical features aligned best to the features of the 4-OHT SB pharmacophore model. Moreover, a strong correlation between pharmacophore fit scores and docking scores was indicated from its R-squared (*R*^2^ = 0.75).

### 2.3. Interpretation of Molecular Docking Results and Pharmacophore Modeling

**Figure 6:**
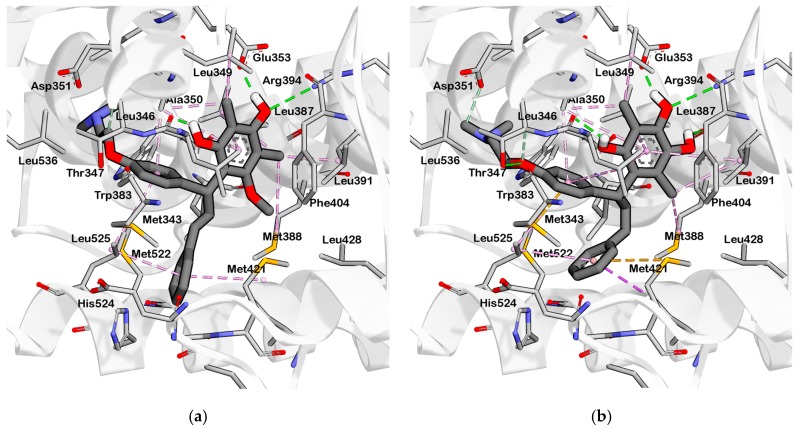
Interaction of (**a**) HNS9 and (**b**) NHS10 within binding site of ERα. Hydrogen bond, ion-ion interaction, and pi-alkyl interactions are represented in green, blue and purple colored dashed lines, respectively.

HNS9 and HNS10 displayed the best pharmacophore fit scores compared to the other derivatives and ∆G free energy of binding (−12.01 kcal/mol and −12.33 kcal/mol, respectively) better than 4-OHT (−11.04 kcal/mol). HNS9 formed ten hydrophobic contacts with Met421, Leu525, Met343, Asp351, Thr347, Ala350, Leu349, Leu391, Leu384, and Met388, and four hydrogen bonds with Leu346, Glu353, Arg394, and Leu387 (Figure 6a), whereas HNS10 formed eight hydrophobic contacts with Met421, Leu525, Thr347, Ala350, Leu387, Leu349, Met388, and Leu391, and three hydrogen bonds with Leu346, Glu353, and Arg394.

Both HNS9 and HNS10 formed hydrogen bonds with Leu346, Glu353, and Arg394 (Figure 6b). The formation of hydrogen bonds with Glu353 and Arg394 is essential for binding to ERα [17], which is in agreement with the previous docking studies involving chalcone [18]. Hydrophobic interactions also contributed to the overall binding of ChalcEA derivatives with ERα. The aromatic rings of HNS9 and HNS10 formed CH-pi hydrophobic interactions with Ala350, Met421, and Leu525 (Figure 6).

**Figure 7:**
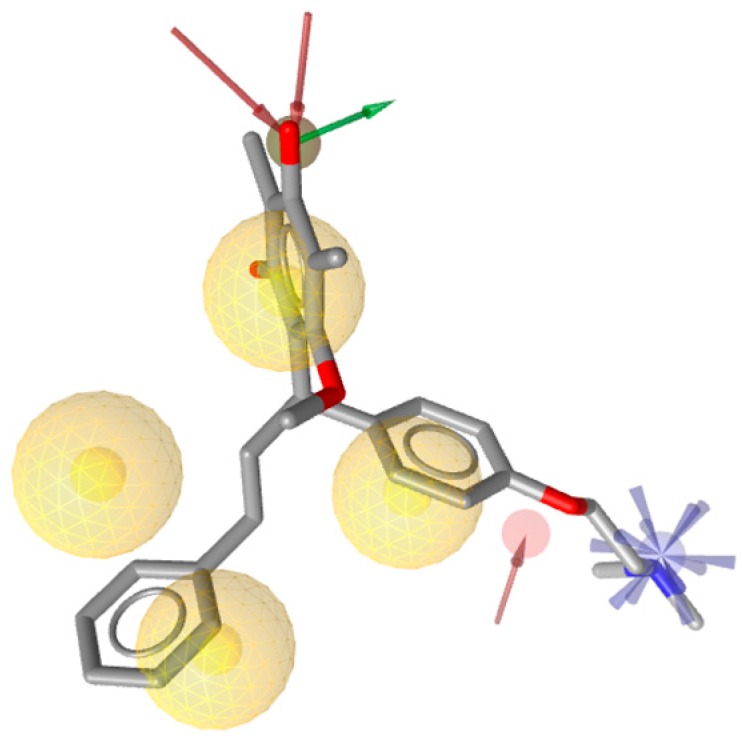

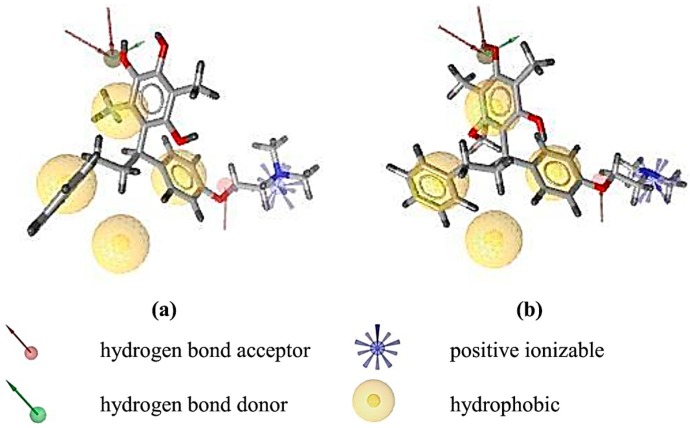
Fit of the (**a**) HNS9 (**b**) HNS10 to the structure-based pharmacophore model derived from 4-OHT with ERα from PDB code: 3ERT. The models were generated using LigandScout 4.1 Advanced. Virtual screening was performed leaving at least two features out. The ligands fit six of the eight features and all of the excluded volumes.

Figure 7 shows that the hydroxyl groups in ortho position on HNS9 hindered the complete mapping with all four hydrophobic features of 4-OHT (Figure 7a). In contrast, the meta position of hydroxyl groups on the aromatic ring of HNS10 enabled a better alignment with the center of the hydrophobic features, thus resulting in a higher fit score (Figure 7b).

## 3. Discussion

To address the issue related the agonistic effect of 4-OHT in the uterus [19], Elwood et al. reported that a shorter distance between Asp351 would likely to decrease the agonistic effect of 4-OHT to uterus [20]. We measured and compared the distances of the dimethylaminoethoxy groups of 4-OHT, HNS9, and HNS10 to be 3.3 Å, 3.2 Å, and 2.8 Å respectively, indicating that HNS9 and HNS10 might form stronger interactions with Asp351 and potentially a decreased agonistic effect in the uterus. However, further theoretical studies related to the agonistic effects of 4-OHT would need to be conducted to verify the hypothesis that the modified ChalcEA might not have the same adverse effects as observed with tamoxifen. Future studies will elaborate the synthesis and biological evaluation of the best predicted ChalcEA derivatives to assess their interaction with ERα and their potential for modulating anti-breast cancer activity.

## 4. Materials and Methods

**Figure 8:**
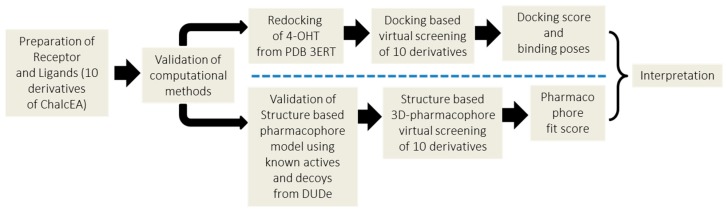
The general scheme of methodologies in the present work.

The general scheme of methods in this work is presented in Figure 8.

### 4.1. Molecular Docking Simulation

The positive control system was the X-ray crystallography derived ERα in complex with 4-OHT taken from Protein Data Bank (PDB ID: 3ERT) [21]. The macromolecule and ligand structures were separated using Discovery Studio Visualizer 4.0. 3D structures of ligands were prepared and optimized by Hyperchem (HyperCube, Inc., Gainesville, FL, USA) and LigandScout 4.1 Advanced (Inte:Ligand GmbH, Vienna, Austria). The molecular docking simulation methods were carried out according to a previously reported study [13]. All ligands and the ERα receptor were prepared for docking using AutoDockTools (ADT) 1.5.6. The ligands and receptor were protonated. The default Kollman charges [22] and solvation parameters were allocated to the protein atoms. Gasteiger charges were added to each ligand atom [23]. A grid box comprised of 40 × 40 × 40 points spaced by 0.375 Å was centered on the ERα active site (x = 30.282, y = −1.913, and z = 24.207). The pre-calculated binding affinity of each ligand’s atom type was prepared using Autogrid [24].

AutoDock 4.2 was utilized for the molecular docking simulation. The parameters of the Lamarckian Genetic Algorithm (LGA) were: 100 runs, elitism of 1, the mutation rate of 0.02, the population size of 150, a crossover rate of 0.80 and 5,000,000 energy evaluations [25]. The resulting docked conformations were clustered using a root-mean-square deviation (RMSD) tolerance of 1.0 Å. The ligand conformation with the lowest free energy of binding (ΔG), chosen from the most favored cluster, was selected for the further analysis. The docking results were visualized using Accelrys Discovery Studio Visualizer 4.0, and the ligand-interaction features for each pose within the binding pocket were determined automatically using LigandScout Advanced 4.1.

### 4.2. Structure-Based 3D-Pharmacophore Modeling

A 3D SB-pharmacophore model was derived automatically from the X-ray derived structure of ERα in complex with 4-OHT, (PDB code: 3ERT) using Ligandscout 4.1 Advanced [15]. The resulting 3D-interaction feature model was validated for its ability to distinguish true active compounds from decoys by screening a set of 626 known active and 20,773 decoy compounds obtained from the enhanced Database of Useful Decoys (DUDe: http://dude.docking.org) [26]. The libraries from DUDe were converted into 3D multi-conformational databases for virtual screening using the LigandScout 4.1 Advanced algorithm, idbgen, which computes conformations and annotates each conformation with pharmacophore features. All of the ChalcEA derivatives were screened virtually using the validated 3D-SB pharmacophore model and the LigandScout 4.1 Advanced VS algorithm, iscreen with a maximum of 2 omitted features to identify and rank ligands in the library that could fit the geometry and features of the 3D-model. The pharmacophore fit score measured the fit of features of each hit compound to the pharmacophore model features and was used to rank the hit molecules retrieved by the pharmacophore model.

## 5. Conclusions

Essential interactions of ChalcEA derivatives with ERα involves hydrogen bond and hydrophobic non-covalent features. The best derivatives among the ten designed candidates identified using the molecular docking and pharmacophore modeling studies were HNS9 and HNS10. HNS9 contained a modification at R1 (methyl), R2 (hydroxyl), R4 (hydroxyl) and R5 (dimethylaminoethoxyphenyl), while HNS10 only at the R5 with a similar group with HNS9. Both HNS9 and HNS10 still complied the Lipinski’s Rule of Five. The free energy of binding (ΔG) of HNS9 and HNS10 were −12.15 kcal/mol and −12.33 kcal/mol, respectively. Both contained key interaction features that could be geometrically aligned with a validated SB pharmacophore model of 4-OHT (pharmacophore fit score of 67.07). The two derivatives represent rational computationally designed compounds prioritized for further biological investigation and hit optimization targeted for future development of chalcone based derivatives as new anti-breast cancer therapies with ERα inhibitor activity and better side effect profiles compared to tamoxifen.

## Figures and Tables

**Table 1:** Derivatives of 2′,4′-dihydroxy-6-methoxy-3,5-dimethylchalcone (ChalcEA).

ChalcEA 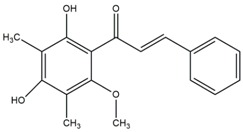			ChalcEA Derivatives (HNS1-10) 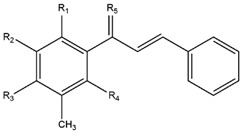			
No.	Molecule Name	R1	R2	R3	R4	R5
1	HNS1	OCH_3_	H	H	H	-
2	HNS2	H	H	H	H	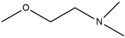
3	HNS3	H	H	H	H	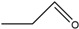
4	HNS4	H	H	H	H	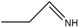
5	HNS5	H	H	H	H	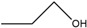
6	HNS6	H	H	H	H	-
7	HNS7	CH_3_	H	-	H	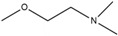
8	HNS8	H	H	H	H	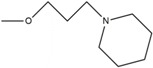
9	HNS9	CH_3_	OH	H	OH	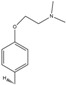
10	HNS10	-	H	-	H	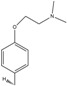

**Table 2:** Computed standard properties of ChalcEA derivatives based on Lipinski’s Rule of Five. HB indicates hydrogen bond.

No.	Derivative Name	Molecular Weight	Log P	Number of HB Donors	Number of HB Acceptors
1	HNS1	284.355	3.60	1	3
2	HNS2	374.501	2.27	3	4
3	HNS3	328.408	4.03	2	4
4	HNS4	330.484	3.07	3	3
5	HNS5	330.424	3.82	3	4
6	HNS6	399.531	4.22	2	4
7	HNS7	372.529	2.88	2	3
8	HNS8	427.585	5.00	2	4
9	HNS9	436.572	3.75	4	4
10	HNS10	450.599	4.05	3	4

**Table 3:** Results of molecular docking simulation of ChalcEA derivatives in ligand binding domain of estrogen receptor alpha (ERα).

No.	Molecule Name	Chemical Formula	∆G (kcal/mol)	Number in Cluster	Calculated Ki (nM)	Interactions with Amino Acids	
						Hydrogen Bond	van der Waals (Hydrophobic)
1	HNS1	C_18_H_18_O_3_	−9.30	35	153.41	Glu353, Arg394	Leu525, Met421, Leu387, Ala350, Leu349
2	HNS2	C_22_H_29_NO_4_	−9.25	29	66.92	Leu346, Leu387	Leu391, Met388, Leu384, Leu525, Met421, Met343, Asp351, Thr347
3	HNS3	C_22_H_29_NO_4_	−9.01	64	249.32	Gly420	Gly521, Val418, Leu346, Met343, Met421, Leu525, Ala350, Thr347, Trp383, Leu387, Leu391, Asp351, Leu348
4	HNS4	C_20_H_23_NO_3_	−9.26	53	162.64	Thr347, Leu346, Glu353, Arg394	Leu349, Ala350, Leu387, Leu391, Met388, Met421, His524, Leu525
5	HNS5	C_20_H_24_O_4_	−9.44	75	120.52	Thr347, Leu346, Glu353, Arg394	Leu387, Ala350, Leu349, Leu391, Met388, Met421, Leu525
6	HNS6	C_24_H_31_NO_4_	−9.76	30	70.51	Leu346, Glu353, Arg394	Leu387, Leu349, Leu391, Ala350, Leu525
7	HNS7	C_23_H_31_NO_3_	−9.93	49	52.67	Arg394, Leu387, Thr347	Leu346, Leu349, Ala350, Asp351, Leu525, Met421, Met388, Leu391
8	HNS8	C_26_H_35_NO_4_	−10.96	43	9.27	Glu353	Met421, Leu525, Met388, Leu391, Leu346, Leu349, Leu387, Ala350, Thr347, Trp383
9	HNS9	C_27_H_31_NO_4_	−12.15	83	1.25	Leu346, Glu353, Arg394, Leu387	Met421, Leu525, Met343, Asp351, Thr347, Ala350, Leu349, Leu391, Leu384, Met388
10	HNS10	C_28_H_33_NO_4_	−12.33	42	0.91	Leu346, Glu353, Arg394	Met421, Leu525, Thr347, Ala350, Leu387, Leu349, Met388, Leu391

**Table 4:** LigandScout pharmacophore fit score of ChalcEA derivatives retrieved using the 3D-structure-based pharmacophore derived from 4-hydroxytamoxifen (4-OHT) bound to the estrogen receptor alpha (ERα). A higher fit score indicates a better geometric alignment of the features of the compound to the 3D-pharmacophore model. The docking score of each compound is provided for comparison.

No.	Compound	Pharmacophore-Fit Score	Docking Score (kcal/mol)
1	ChalcEA	45.90	−8.23
2	HNS1	46.76	−9.30
3	HNS2	46.62	−9.25
4	HNS3	56.57	−9.01
5	HNS4	46.61	−9.26
6	HNS5	56.68	−9.44
7	HNS6	55.33	−9.76
8	HNS7	55.00	−9.93
9	HNS8	56.20	−10.96
10	HNS9	66.50	−12.15
11	HNS10	67.07	−12.33
